# Report of a General Plan for the Promotion of Public and Personal Health, Devised, Prepared and Recommended by the Commissioners Appointed under a Resolve of the Legislature of Massachusetts, Relating to a Sanitary Survey of the State

**Published:** 1851-04

**Authors:** 


### BIBLIOGRAPHICAL NOTICES.

*Report of a General Plan for the promotion of Public and Personal Health, devised, prepared and recommended by the Commissioners appointed under a resolve of the Legislature of Massachusetts, relating to a Sanitary Survey of the State. Presented April 25th,* 1850. Boston, 1850. For sale by Lindsay & Blakiston, Philadelphia.

An English reviewer, alluding to the great number of works on hygiene, has observed, that "sanitary publications are now 'as plenty as blackberries.'" While this quaint remark may be apposite to the science of health abroad, it has neither force nor meaning on this side the Atlantic.

On the Continent, especially in France and the German and Prussian States, sanitary and medical police are under the control of government, and have been applied extensively to society. In England, within the last five and twenty years, sanitary improvement has become a popular question. It is one of the great questions of the day. Men of science, physicians, philanthropists, and members of parliament, are fully aroused to a sense of its importance, and are lending both their public and private influence to favor its advancement. Health associations have been organized, and the members are urging their claims upon the people with a zeal well becoming the intrinsic merit of the sanitary question.

With us, however, the case is widely different; sanitary publications being rare. It is only of late years, that the subject has created any interest whatever. Here and there, beyond the pale of the medical profession, may be found a solitary advocate for the science of Public Health; but, with the masses, the whole question is almost, if not altogether void of interest. With the medical profession at large, we might also say with truth, it is an "unexplored region of medical inquiry," notwithstanding it embodies principles and facts, intimately interwoven with the preservation and invigoration of human life, as well as the removal of many of the productive causes of disease and death.

By our public authorities, the laws of health and life, of happiness and usefulness, have been shamefully neglected; nor will the well-timed animadversion of Dr. J. Curtis on the acts of the Legislature of Massachusetts, in his report on Public Hygiene, read before the American Medical Association, at its sitting in May 1849, apply with less force to the government of this and every other state in the confederacy:—"We have legislated, *usque ad nauseam*, on almost everything but that which concerns us most, namely, the surest sources of health and life, and consequent happiness and prosperity. By legislation, we have protected the beasts of the field, the fowls of the air, and the fish of the sea; by legislation, we have encouraged the arts and sciences, except those which would most directly enable us to live long, useful and happy; by legislation we have granted privileges to the manufacturer, developed the resources of the agriculturist * * * * * * * all of which, is most praiseworthy; but we have neglected to use the means, for securing attainable longevity and exalted happiness, as well as some of the most prolific sources of state wealth." There is more truth than romance in this expressive extract, and it is deeply to be lamented, that the study of life-knowledge, one of the most sublime subjects that can engage the human intellect, should be so little regarded by the wise and the good.

We may congratulate ourselves, however, that a brighter day is dawning upon us, in this department of medical science. Recently, sanitary reform has been the subject of scientific investigation in Massachusetts, and several valuable tracts have issued from the press, urging the claims of Hygiene, and calculated to exert a salutary influence. In our own state, the College of Physicians has been earnestly engaged in prosecuting the science, and during the last year, a most interesting and elaborate report from the pen of Dr. John Bell, Chairman of the Standing Committee on Public Hygiene, has been published in the summary of its transactions. The public is indebted, however, for the first organized movement, on an extensive scale in this country, in behalf of sanitary reform, to the American Medical Association. At the first session of this learned body in 1848, a communication was laid before it, from the Medical Department of the National Institute at Washington, directing its attention to the science of Hygiene. A committee of twelve was appointed to report on the subject, and at the meeting the following year, most valuable, and with us "new contributions to science" were presented, embodying a mass of information intimately connected with the causes of disease and death, thus opening a vast field for medical research never before occupied to any extent by the profession in this country, and laying the foundation for further and more enlarged prosecution. Among the states, Massachusetts has been the first to move in this department of medical literature; nor do we require any better evidence of the interest that the authorities of the "old Bay State" have felt in sanitary reform, than the publication of the admirable work, the title of which stands at the head of this article.

The suggestion for a sanitary survey of the state of Massachusetts originated with their medical society and the American Statistical Association. The legislature, in compliance with their petition, authorized the Governor and council "to appoint three persons to be commissioners, to prepare and report to the next General Court, a plan for a sanitary survey of the state, embracing a statement of such facts and suggestions, as they may think proper to illustrate the subject." The Commissioners appointed, were Lemuel Shattuck, Esq., of Boston, Nathaniel P. Banks, Jr., Esq., of Waltham, and Dr. Jehiel Abbott, of Westfield.

From the well known reputation of the first named gentleman, as an eminent statist, and his perfect familiarity with the laws of public health and their practical operation, although not a physician, the whole preparation of the work was committed to his hands, and we are informed, that all its plans and features, together with the accompanying bill for legislative enactment, are the result of his undivided labor. Our thanks are certainly due to Mr. Shattuck, for the very able and complete manner in which he has managed to throw together such an amount of sanitary information as is contained in this volume.

The book opens with an exposition of what is intended by a sanitary survey of the state, to wit:—"an examination or survey of the different parts of the Commonwealth,—its counties, its towns, and its localities,—to ascertain the causes which favorably or unfavorably affect the health of its inhabitants." Then follows an interesting account of the sanitary movement, both abroad and at home, beginning with the earliest records of scripture history, when, in the garden of Eden, the Supreme Lawgiver, pronounced the first sanitary law, "in the day that thou eatest thereof, thou shalt surely die,"—and bringing it down through succeeding ages, to the present time. The research and labor, necessary for gathering the materials to be found in this department of Mr. Shattuck's work, are fresh evidences of his untiring zeal and industry, in the cause of public health.

Our space, will not allow of an analysis of this succinct history of Hygiene, which has been derived from various authentic documents, and it is, perhaps, the less necessary, as we feel assured, that those who take a lively interest in the science of health, as well as those who are seeking information of the progress and usefulness of sanitary reform, will desire to obtain for themselves a copy of the work. To be properly appreciated in all its important bearings, this richly stored volume must be read entire.

The statistical tables, compiled from the Registration reports of Massachusetts, and drawn up with great accuracy, convey much valuable information, and exhibit the results of certain etiological influences, connected with locality, climate, pursuits and habits, both moral and physical, on human health and longevity. Among other results exhibited by these statistics, is, that the vital energies of man are decreasing, "and that the average duration of life is not as great now as it was forty or fifty years ago." This startling fact, has been confirmed by recent investigations in England, and our author believes, that it must be attributed to the effects of imperfect sanitary laws, and hence, if there is an improvement in these laws, there will be a corresponding improvement in the condition of human health and longevity.

Vital statistics carry with them, some of the most important facts and phenomena connected with the prosperity and healthy condition of a city or state. Could they be gathered in a uniform manner, throughout our entire country, and controlled by legislative enactment, as has been proposed by the American Medical Association, there is no estimating the happy results, in a sanitary point of view, that would be realized in a few years.

Under the third division of subjects embraced in this "Sanitary Commission," we have the outline of a "plan for a sanitary survey of the state," which, in the clear language of the author, "Consists of a series of measures, which may be rendered permanent if desired, presented in the form of separate recommendations. They are divided into two classes, and are to be regulated and controlled by agencies which are proposed to be established; one by the legislative authority of the state, and the municipal authorities of towns and cities, and the other, by social organization and personal action."

Under the first division, the author includes an entire revision of the Health Laws, and the establishment of a general Board of Health for the state, and subordinate or local boards; "a census; a registration of births, marriages and deaths; atmospheric observations; causes of disease and death; laying out of new towns; regulation of public buildings; manufactories and private dwellings; over crowded lodging houses and cellar dwellings; public squares and ornamental trees; mill-ponds and stagnant water; house to house visitation; observations on sickness in general, and in schools; vaccination; consumption; nuisances; evils of intemperance; inquests; insane and idiots; interments; emigrant ships and steamers; quarantine and sanitary evils of emigration, &c."

Under the second division, embracing regulations which may be adopted without the aid of legislative enactment—are, "sanitary associations; tenements for the laboring classes; public baths and wash-houses; refuse of towns; abatement of smoke nuisance; physician's and apothecaries' prescriptions; adulterated food and drugs; education of nurses; education in sanitary science; physician's records of cases; clergymen interested in public health; sanitary observations in families and personal sanitary examinations."

The report closes with many well-ordered and intelligent "reasons for approving the plan recommended," together with a sensible appeal in its favor to the denizens of the state. Take it altogether, this report is a valuable acquisition to our medical literature, and while it embraces a very magnificent and comprehensive scale of operations, we are not surprised that the author should express some doubt of its entire sanction by those for whose special benefit it was prepared, unless it has "a careful consideration before judgement is passed upon it, and when so considered, we have great confidence that we shall have the approval of all candid minds."

Nor can we believe it to be entirely practicable (although it may have, as doubtless it will, the approval of every intelligent and candid mind,) to cultivate at once, the immense fields of sanitary improvement, contemplated in the report. We apprehend, too, that there are many difficulties to grapple with, even in Massachusetts, where the public mind is really in advance of other states on this subject, before the recommendations of the commission, involving as they do, so many features and plans entirely new, can be complied with. But we would offer no discouragement; our principle is, "expect great things, attempt great things," and though the entire classes for survey named in the plan, should never be completed, enough will no doubt be accomplished to work important changes and results in the etiological character and prevention of disease, and in the moral and physical condition of the human race.

Accompanying this report, is the "Bill drawn by the Commission and recommended to the Legislature for enactment." The main features in this bill, consist in "the establishment of a Central General Board of Health or the whole state, and a local Board of Health for each city and town in the state; each to be composed of competent men, who are to have the general superintendence of all matters relating to the public health, within their respective jurisdictions." Among other requirements embraced within the duties of the general board, they are to "advise the state as to the location and erection of public buildings, and as to the sanitary regulations of public institutions," they are to prepare an annual report for the legislature, embracing "such remarks, as their observation, experience and reflection may suggest, as to the sanitary condition of the state, its institutions and inhabitants; and recommend the adoption of such useful sanitary measures as in their judgement may lead to improvement." They are also authorized to expend fifty dollars annually for books suitable for a sanitary library. They are to "superintend each enumeration of the inhabitants of the state," besides many other important and necessary duties, entirely new with boards of health as at present organized.

The powers to be conferred upon the general Board of Health and on Local Boards, by the enactment of the bill proposed by the Commission, are ample in their nature and design. The bill has been drawn up with great care, after much reflection and investigation, embracing every subject matter involved in the great sanitary question. Should it be passed by the General Court of Massachusetts so as to become a law, in its present shape, we have no hesitation in promising that the health, wealth, and prosperity of the state will be eminently improved. Besides the conferring of such blessings on the state, the adoption of the recommendations for a sanitary survey and the passage of the Health act appended thereto, would give an impetus to the authorities of other states to "go and do likewise."

Thus would the noble example of "the forefathers land" be imitated, her influence be felt the wide country over, and the adoption and administration of wise sanitary laws prove a benefit and a blessing for generations to come. God speed the day when the ignorant on this vital question may be instructed, when life-knowledge, or the art of preserving life, by invigorating health and removing the causes of disease, shall take its place side by side, with the progressive sciences, thus meeting the demands of the spirit of the age.

Attached to the report is a copious appendix, illustrating, by a variety of well written articles, many of which, however, have appeared in print elsewhere, the principles and practice of sanitary reform as inculcated throughout the book.

In concluding this cursory notice of Mr. Shattuck's highly interesting work, it only remains for us to express the pleasure we have derived from its perusal. We heartily commend it to the careful reading of every physician, philanthropist, political economist, statistician and statesman, as embracing rich materials out of which may be formed correct principles of public and private Hygiene. Our sincere desire is, that it may be widely circulated, and contribute largely to the further developement of an already too much neglected science; one that cannot be overrated, and that is essential both to the health and happiness of the million.

